# Pharmacokinetic–Pharmacodynamic Modeling of Enrofloxacin Against *Escherichia coli* in Broilers

**DOI:** 10.3389/fvets.2015.00080

**Published:** 2016-01-07

**Authors:** KaNa Sang, HaiHong Hao, LingLi Huang, Xu Wang, ZongHui Yuan

**Affiliations:** ^1^National Reference Laboratory of Veterinary Drug Residues (HZAU), Huazhong Agricultural University, Wuhan, China; ^2^MAO Key Laboratory for Detection of Veterinary Drug Residues, Huazhong Agricultural University, Wuhan, China; ^3^MOA Laboratory for Risk Assessment of Quality and Safety of Livestock and Poultry Products, Huazhong Agricultural University, Wuhan, China

**Keywords:** enrofloxacin, *Escherichia coli*, PK/PD modeling, broiler, intestinal content

## Abstract

The purpose of the present study was to establish a pharmacokinetic/pharmacodynamic (PK/PD) modeling approach for the dosage schedule design and decreasing the emergence of drug-resistant bacteria. The minimal inhibitory concentration (MIC) of 929 *Escherichia coli* isolates from broilers to enrofloxacin and ciprofloxacin was determined following CLSI guidance. The MIC_50_ was calculated as the populational PD parameter for enrofloxacin against *E. coli* in broilers. The 101 *E. coli* strains with MIC closest to the MIC_50_ (0.05 μg/mL) were submitted for serotype identification. The 13 *E. coli* strains with O and K serotype were further utilized for determining pathogencity in mice. Of all the strains tested, the *E. coli* designated strain Anhui 112 was selected for establishing the disease model and PK/PD study. The PKs of enrofloxacin after oral administration at the dose of 10 mg/kg body weights (BW) in healthy and infected broilers was evaluated with high-performance liquid chromatography (HPLC) method. For intestinal contents after oral administration, the peak concentration (*C*_max_), the time when the maximum concentration reached (*T*_max_), and the area under the concentration-time curve (AUC) were 21.69–31.69 μg/mL, 1.13–1.23 h, and 228.97–444.86 μg h/mL, respectively. The MIC and minimal bactericidal concentration (MBC) of enrofloxacin against *E. coli* (Anhui 112) in Mueller–Hinton (MH) broth and intestinal contents were determined to be similar, 0.25 and 0.5 μg/mL respectively. In this study, the sum of concentrations of enrofloxacin and its metabolite (ciprofloxacin) was used for the PK/PD integration and modeling. The *ex vivo* growth inhibition data were fitted to the sigmoid *E*_max_ (Hill) equation to provide values for intestinal contents of 24 h area under concentration-time curve/MIC ratios (AUC0–24 h/MIC) producing, bacteriostasis (624.94 h), bactericidal activity (1065.93 h) and bacterial eradication (1343.81 h). PK/PD modeling was established to simulate the efficacy of enrofloxacin for different dosage regimens. By model validation, the protection rate was 83.3%, demonstrating that the dosage regimen of 11.9 mg/kg BW every 24 h during 3 days provided great therapeutic significance. In summary, the purpose of the present study was to first design a dosage regimen for the treatment *E. coli* in broilers by enrofloxacin using PK/PD integrate model and confirm that this dosage regimen presents less risk for emergence of floroquinolone resistance.

## Introduction

Antimicrobial resistance is a principal threat to both animals and humans, the main reason for the increasing bacterial resistance is the overuse and misuse of antibiotics. Optimizing the dosage schedules is essential for achieving clinical cures and minimizing the emergence of antimicrobial drug resistance (1, 2). Currently, the optimal dose regimen should be calculated according to the relationship between pharmacokinetics (PKs) and pharmacodynamics (PD) (3). PK/PD modeling can reflect the relationship between the drug, the bacteria, and the animals. It has been demonstrated in many studies that the integration and modeling of the relationship between PK and PD can quantify the potency and efficacy of antimicrobials against target pathogens and provide a basis for the dose optimization (4–7).

PK/PD modeling can be used to evaluate the clinical efficacy of antimicrobial agents (4, 8). The most widely used PK parameters are the area under the plasma concentration-time curve (AUC) and the maximum plasma concentration (*C*_max_). The most commonly used PD parameters include minimal inhibitory concentration (MIC) and time-kill curve. The integration of PK and PD establishes a mathematical and theoretical link between them. The three PK/PD parameters are AUC/MIC ratio, *C*_max_/MIC ratio, and the percentage of a 24-h time period that the unbound drug concentration exceeds the MIC (T > MIC) (7, 9, 10). Fluoroquinolones are classified as typical concentration-dependent drugs, the initially reported AUC/MIC and *C*_max_/MIC are better predictors than T > MIC for the antibacterial effect (11, 12). Therefore, it is very important to determine the AUC/MIC value of enrofloxacin against *Escherichia coli* in broilers.

*Escherichia coli* is a Gram-negative facultative anaerobe, it is a frequent cause of septicemia, Enterocolitis, and diffuse peritonitis. Colibacillosis has caused great economic losses to the poultry industry and commonly fluoroquinolones are used for the treatment (13–15). At the same time, the serious abuse of fluoroquinolones in clinical settings has been implicated in treatment dissatisfaction or even treatment failure, it also contributes to the emergence of bacterial antibiotic resistance.

Pharmacokinetics of enrofloxacin has already been studied in many species, including calves, turkeys, horses, goats, sheep and pigs (16–21); however, studies on the PK of enrofloxacin in infected animals are quite few even though the PK parameters in infected animals are more close to the clinical conditions (22). The metabolism of enrofloxacin to ciprofloxacin in broilers is limited to 5 and 10% (23, 24). There is some research on the PK of enrofloxacin in intestinal contents (25, 26), but limited data are available regarding the PK/PD in broiler intestinal contents.

The objectives of the study were to: (1) investigate the population MIC and select clinical strains with higher pathogenicity for establishing bacterial infected model, (2) determine the PK of enrofloxacin orally administrated in healthy and infected broilers, (3) determine the PD of enrofloxacin against clinical *E. coli* pathogenic strains, (4) use the PK/PD model to optimize the dosage regimen of enrofloxacin against colibacillosis in broilers, and (5) validate the model to verify the clinical efficacy of the resulting dosage regimen.

## Materials and Methods

### Animals

#### Broilers

Thirty-day-old healthy KeBao broilers obtained from a commercial poultry farm in Jiang Xia were utilized for this experiment (*n* = 160), the broilers were mixed sex (50:50) with weights ranging between 0.9 and 1.2 kg. The broilers were randomly distributed into two treatment groups (*n* = 80/treatment group) Group 1 served as the non-infected healthy controls animals and Group 2 the *E. coli-*infected disease animals. All broilers were allowed 7 days for acclimation before the experiments began. All broilers were housed in a climate control environment under identical conditions with standard commercial diet and water supplied.

#### Mice

Four-week-old healthy Kunming mice (SPF grand, kw: 18 ± 2 g, *n* = 120, half male and half female) were obtained from the Center of Experimental Animal of Hubei, and housed in SPF animal room in our lab [Licence# SCXK(E) 2008-0005].

All experimental procedures in this study were performed according to the guidelines of the committee on the use and care of the laboratory animals in Hubei province, China. The study was approved by the Animal Care Center, Hubei Science and Technology Agency in China (SYXK 2013–0044). All the animals were monitored throughout the study for any signs of adverse effects.

### Chemicals and Reagents

The Enrofloxacin reference standard (98% purity) and Ciprofloxacin Hydrochloride reference standard (95% purity) were purchased from Dr. Ehrenstorfer (Augsburg, Germany). The Enrofloxacin API (98% purity) used for PKs study was acquired from a local distributor (Wuhan Midland Chemical Co., Ltd.), the supplied powder was dissolved in 5% sodium carboxymethyl cellulose (CMC) to allow for oral administration. Acetonitrile (ACN), formic acid and methanol (MeOH) were provided by TEDIA (USA). All chemicals used in this experiment were analytical grade or higher and dissolved or diluted using de-ionized water (Milli-Q Millipore Corp.).

### Bacteria

The clinical *E. coli* pathogenic strain designated Anhui112 was previously isolated from broiler chickens with colibacillosis. This strain was used for the PD study and the *E. coli* infection model. The commonly used quality control standard *E. coli* strain ATCC 25922 was purchased from Chinese Veterinary Culture Collection. All strains were stored at −80°C until use. Before each experiment, these bacteria cultures were sub-cultured on Mueller–Hinton agar and incubated at 37°C for 18–24 h.

### Determining the MIC of Enrofloxacin Against Clinical *E. coli* Isolates

A total of 929 clinical isolates collected from five provinces in central China and stored in the laboratory were studied in this experiment. The determination of MIC for enrofloxacin and ciprofloxacin against all clinical *E. coli* strains was determined by the microdilution broth method, using the *E. coli* ATCC 25922 as the experimental control strain and following CLSI (Clinical and Laboratory Standards Institute, 2009, M07-A9) protocol. MICs were found to be in the range between 0.04 and 64 μg/mL. Using SPSS 16.0, the 50% minimum inhibitory concentration (MIC_50_) 0.50 μg/mL and 90% minimum inhibitory concentration (MIC_90_) 16.21 μg/mL were determined to represent the population PD of enrofloxacin against clinical *E. coli*.

### Serotype Identification

The clinical *E. coli* strains, approximately 101 strains, with the MIC closest to MIC_50_, were further tested for determining which strains were serotype O or K positive. The O and K anti-serum was purchased from China Institute of Veterinary Drug Control (IVDC). Serotype was determined by traditional slide agglutination test using the commercialized antigen diagnostic serum following previously published methods (27).

### Pathogenicity Experiment on Mice

The strains previously identified as either serotype O or K (*n* = 13) were used in a pathogenicity experiment on mice. Each of the 13 strains were administered intra-peritoneally (200 ul) to a separate group of mice (*n* = 9/group) at three different doses [1 × l0^8^, 1 × l0^7^, and1 × l0^6^ colony forming units (CFU)/mL, *n* = 3/dose] to establish the infection model; additionally there was a none treated control group for a total of 14 groups. All mice were closely observed for the first 6 h and then once every 2 h for the remainder of the day. Any behavioral changes observed were noted and recorded by time and group. At the conclusion of this experiment, the data were analyzed and the most virulent strain was selected to conduct PD study and establish the *E. coli* infection model.

### Establishing *E. coli* Infection Model in Broilers

After a 7-day acclimation period, 160 broilers were randomly selected and distributed into one of two experimental groups (*n* = 80/group). Each of the 80 broilers in the infection group was inoculated by oral gavage with 8 mL of *E. coli* culture (previously determined to be the most virulent) containing 1.2 × 10^9^ CFU/mL. Birds were observed after inoculation for clinical symptoms and pathological changes. After 12 h, sterile tissue (liver) samples were collected by necropsy from dead broilers and cultured to confirm that mortality was caused by the inoculated strain.

### Pharmacokinetic Experiment

After a 7-day acclimation period, 160 broilers were randomly divided into two groups (*n* = 80/group): the *E. coli* infection model and healthy control model. On the morning of the eighth day, enrofloxacin was orally administered at a dose of 10 mg/kg to all broilers. At the following time points after oral administration of enrofloxacin: 0, 0.25, 0.5, 0.75, 1, 1.5, 2, 3, 4, 8, 12, 24, and 48 h, six broilers per group were humanly killed to collect blood samples (2 mL) and intestinal contents (2 mL).

Blood samples collected with anticoagulant were centrifuged for 10 min at 3000 rpms to obtain plasma. The plasma was removed to a clean sterile tube and stored at −20°C until analysis. At a later time point, 1 mL of plasma was added to a 10 mL tube, 2 mL of ACN was then added to precipitate proteins. The tube was vortexed for 2 min and centrifuged at 4000 rpms for 10 min, the aqueous phase was transferred to a clean tube and dried under nitrogen. One milliliter of 14% ACN was used to reconstitute the dried pellet. The sample was filtered through a 0.22 μm membrane into the high-performance liquid chromatography (HPLC) detection vial.

Intestinal content (1 g) was homogenized for 1 min in a 50 mL tube, 2 mL of 0.5 M EDTA (pH 7.0) was then added to the homogenate followed by 15 mL of dichloromethane. The mixture was vortex-mixed for 1 min and centrifuged at 5000 rpms for 5 min. This extraction procedure was repeated twice and then dried under nitrogen. The dried pellet was reconstituted using 1 mL 12% ACN then centrifuged at 12,000 rpm for 5 min before analysis. The supernatant was removed and filtered through a 0.22 μm membrane, into the HPLC detection vial.

The concentration of enrofloxacin and ciprofloxacin in plasma and intestinal content were determined by the HPLC method with UV (UltraViolet) detector (28) under the following conditions: UV detection 278 nm, column temperature 30°C, mobile phase water with 0.1% formic acid and ACN, and sample size 40 μL injected into the HPLC (Waters 2695) system with a flow rate 1 mL/min.

The concentration–time data variables and Pharmacokinetics parameters of enrofloxacin and ciprofloxacin were derived from the Winnonlin program (version 5.2.1, Pharsight Corporation, Mountain View, CA, USA). The plasma concentration determined data were analyzed with compartmental method. The linear trapezoidal rule was used to calculate the AUC.

### Determination of MIC, MBC, MPC, and PAE

The MIC of Anhui112 was determined using the previous detailed method.

The minimal bactericidal concentration (MBC) was determined after MIC reading, 100 μL bacteria suspension from each well of the 96 microwell plate was spread on MH agar plates and incubated at 37°C for 24 h for bacterial counting. MBC in broth and intestine were determined as the lowest concentration at which bacteria numbers were reduced by 99.9%.

The mutant prevention concentration (MPC) of enrofloxacin was determined using the agar method (29). The 10^10^ CFU/mL *E. coli* strains were inoculated on the agar plates containing serial concentration of enrofloxacin (1 × MIC, 2 × MIC, 4 × MIC, 8 × MIC, 16 × MIC, and 32 × MIC) and cultured at 37°C for 72 h. The MPC was defined as the lowest concentration of enrofloxacin on agar plates without bacterial growth.

For the post-antibiotic effect (PAE) determination, the bacterial was exposed to three different concentrations (1 × MIC, 2 × MIC and 4 × MIC) of enrofloxacin for 1 or 2 h. The media containing enrofloxacin was removed by centrifuge at 12000 × g for 5 min. The bacterial was re-grew in fresh media without enrofloxacin for another 24 h. The bacterial numbers were determined at different time points. The PAE was the time difference (in hours) for antimicrobial-treated bacterial to increase in number by 1 log_10_ minus the same determination for non-treated cultures of the same test bacterial.

### Drawing *In vitro* and *Ex Vivo* Bacterial Killing Curves

*In vitro* bacterial killing curves were determined in MH broth. Different concentrations of enrofloxacin: 1/2MIC, 1MIC, 2MIC, 4MIC, 8MIC, 16MIC, and 32MIC were prepared in MH broth, the tubes were then inoculated with *E. coli* Anhui112 (10^6^CFU/mL) and incubated at 37°C. The bacterial count (CFU/mL) was determined for each tube after 1, 2, 4, 6, 8,12, and 24 h of incubation. Briefly, 100 μL culture was obtained for each time point, serially diluted with sterile saline plated and the colonies counted the next morning. The limit of detection was 10 CFU/mL. Each concentration test was performed in triplicate.

The *Ex vivo* bacterial killing curves were determined in intestinal content samples obtained from broilers at different time points after oral administration with enrofloxacin following the method as described earlier for the *in vitro* study. The tubes containing bacterial culture and intestinal content were incubated at 37°C and then viable bacteria counts were determined at 1, 2, 4, 8, 12, and 24 h time points. The limit of detection was 10 CFU/mL. Each concentration test was performed in triplicate.

### PK and PD Integration and Modeling

All PK parameters were calculated by WinNonlin 5.2 software, including T > MIC, *C*_max_, T_1/2_, AUC, etc. PK analysis was performed with one-compartmental model. Using *in vitro* MIC and *in vivo* PK parameters, the surrogate parameters (*C*_max_/MIC, AUC/MIC, and T > MIC) of intestinal content were determined after oral administration of enrofloxacin. The relationship between the *ex vivo* AUC_24h_/MIC ratio and the bacterial count after 24 h of incubation in intestinal content was determined by using the sigmoid inhibitory *E*_max_ model. This model is described by the following equation:

$$E=E_{\max}-\frac{(E_{\max}-E_{0})\cdot C^{N}}{C^{N}+EC_{50}{\;}^{N}}$$

In which, *E* is the antibacterial effect measured as the change in the bacterial count (log_10_CFU/mL) in the serum sample after 24 h of incubation compared to the initial log_10_CFU/mL; *E*_max_ is the maximum antibacterial effect determined as the difference of log_10_CFU/mL in sample incubated between 0 and 24 h time point; *E*_0_ is the log_10_ difference of bacterial count in the control sample containing tylosin after 24 h of incubation; EC_50_ is the AUC/MIC producing 50% of the maximum antibacterial effect; C is the AUC/MIC in the effect compartment (the *ex vivo* site, that is serum); and *N* is the Hill coefficient, which describes the steepness of the AUC/MIC-effect curve.

To investigate the effect of different dosage regimens, the PD model describing bacterial growth rate in function of enrofloxacin concentration was combined with the PK model and simulations were performed with mlxplore software (version-1.1.0, Lixoft, Orsay, France).

### Dosage Regiment

Four levels of antibacterial effect of enrofloxacin were quantified from the sigmoid *E*_max_ equation by determining AUC/MIC required for bacteriostatic action (no change in bacterial count after 24 h of incubation, *E* = 0), a 50% reduction of the bacterial count, bactericidal action (a 99.9% decrease in the bacterial count, *E* = 3), and bacterial elimination (the lowest AUC/MIC that produce a 99.99% reduction in the count, *E* = 4) in each intestinal content. The calculation of potential optimal dosage can use the following equation:

$$X=d\times \frac{AUC_{\text{ex vivo}}/MIC_{\text{tested}}}{AUC_{\text{in vivo}}/MIC_{\text{target}}}$$

where *d* is the projected dosage (mg/kg), 10 mg/kg was used in this study. The value of the *ex vivo* AUC_24_/MIC obtained from intestinal content represents bacteriostatic activity, bactericidal activity, and elimination of organisms, respectively. The *in vivo* AUC is obtained from the PK using MIC_50_ as the target MIC in this study.

### Risk Assessment of *E. coli* Resistance

The concentration of enrofloxacin was adjusted to 0, 0.125, 0.25, 0.5, 1, 2, 4, and 8 μg/mL with MH broth and 10^9^ CFU/mL of bacteria were added to each tube; after 2, 4, 6, 8,12, 18, 24,36, and 48 h of incubation 100 μL was removed from each tube and plated on MH agar plates which contained 1 μg/mL enrofloxacin. Any plate containing 100 CFUs or less were not considered to be a drug induced mutation.

### Model Validation of the Dosage Regiment

In order to verify the clinical efficacy of the resulting suggested dosage regimen, 24 broilers with artificially induced colibacillosis were divided into two groups randomly (*n* = 12/group), and administered saline or antibiotic at a previously determined dosage regimen. Clinical symptoms and pathological changes were observed and recorded for 48 h.

### Ethical Statement

All the experimental procedures in this study were performed according to the guidelines of the laboratory care and use committee in Hubei province, China. The study was approved by Animal Ethics Committee of Huazhong Agricultural University and the Animal Care Center, Hubei Science and Technology Agency in China (SYXK 2013-0044). All the animals were monitored throughout the study for any adverse effect signs. This experiment is in line with national regulations regarding animal welfare ethics. Ethical approval number is hzauch 2014-002.

## Results

### MIC Distribution of the Clinical *E. coli* Strains

**Figure 1:**
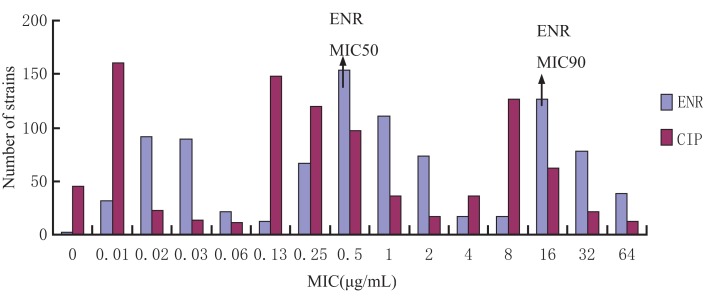
**Enrofloxacin and ciprofloxacin MIC distribution of 929 *Escherichia coli* strains isolated from broiler farms**. The *X*-axis was the MIC values and *Y*-axis was the strain number in each MIC value.

The MIC distribution of the 929 *E. coli* strains to enrofloxacin and ciprofloxacin is shown in Figure 1. The MIC for enrofloxacin and ciprofloxacin exhibited a trimodel distribution, indicating that there may be some resistant strains. The MIC_50_ and MIC_90_ for enrofloxacin were 0.05 and 16 μg/mL, respectively.

### Serotype and Pathogenicity of the Selected *E. coli* Strains

Approximately, 101 *E. coli* strains; which were closest to MIC_50_ (0.05 μg/mL) were selected for further analysis by serotyping specifically selecting for serotypes O and K. Only 13 *E. coli* isolates were identified as O or K serotype. And these 13 *E. coli* isolates were used for a pathogenicity experiment in mice. From the results (Table 1), we were able to identify the most virulent strain, Anhui 112. Therefore, *E. coli* Anhui 112 was selected to conduct the PD study and to establish the infection model.

**Table 1:** **MIC and serotype distribution of clinical isolates (microgram per milliliter)**.

Bacteria	MIC	O	K	Death/total^a^		
				1 **×** l0^8^ CFU^b^	1 **×** l0^7^ CFU^b^	1 **×** l0^6^ CFU^b^
Zhengzhou379	0.25	O75	987P	1/3	0/3	0/3
Zhengzhou384	0.125	O40	K99	1/3	0/3	0/3
Zhengzhou389	0.125	O9	K88	0/3	0/3	0/3
Zhengyang113	0.125	O18	K99	0/3	0/3	0/3
Jingzhou9	0.25	O93	K88	3/3	2/3	1/3
Jingzhou77	0.06	O138	K88	2/3	0/3	0/3
Xinzhou18	0.25	O139	K99	2/3	1/3	0/3
Xinzhou52	0.25	O101	K88	2/3	1/3	1/3
Xinzhou54	0.25	O80	K99	1/3	0/3	0/3
Xinzhou100	0.25	O40	K99	1/3	0/3	0/3
Anhui112	0.25	O2	K88	3/3	3/3	2/3
Zhengzhou16	0.25	O18	K88	1/3	0/3	0/3
Zhengyang18	0.25	O20	K88	0/3	0/3	0/3
Control group				0/3	0/3	0/3

*^a^Represents the death number of mice and the ratio of the total*.

*^b^Number of colonies to each mice by intraperitoneal injection*.

### *E. coli* Infection Model

**Figure 2:**
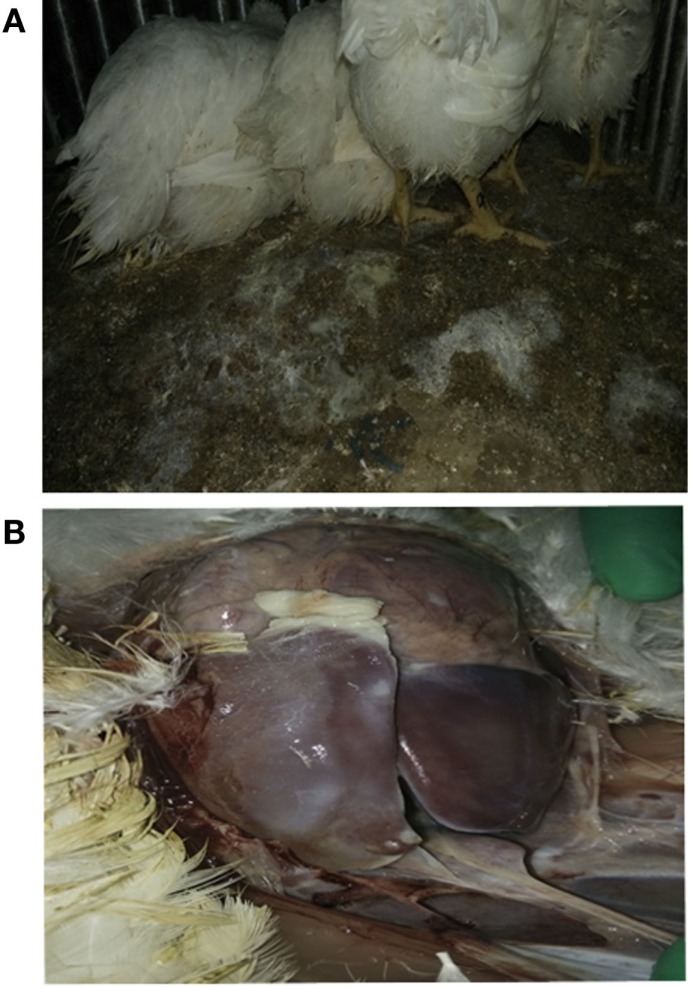
**Broilers infected with *E. coli* Anhui112 strains**. After infection by E.coli, the broilers had serious Diarrhea **(A)** and liver lesions **(B)**.

Using *E. coli* Anhui 112, we were able to successfully reproduce an *E. coli* infection model in commercial broiler chickens, within 12 h after challenge with Anhui 112, broilers exhibited typical clinical signs of colibacillosis, such as the elevated temperature, loss of appetite, and white loose droppings. Lesions examined by necropsy showed a layer of white cellulose pseudo-membranous covering the surface of the liver and heart consistent with classical colibacillosis, clearly seen in Figure 2.

### Pharmacokinetics of Enrofloxacin

**Figure 3:**
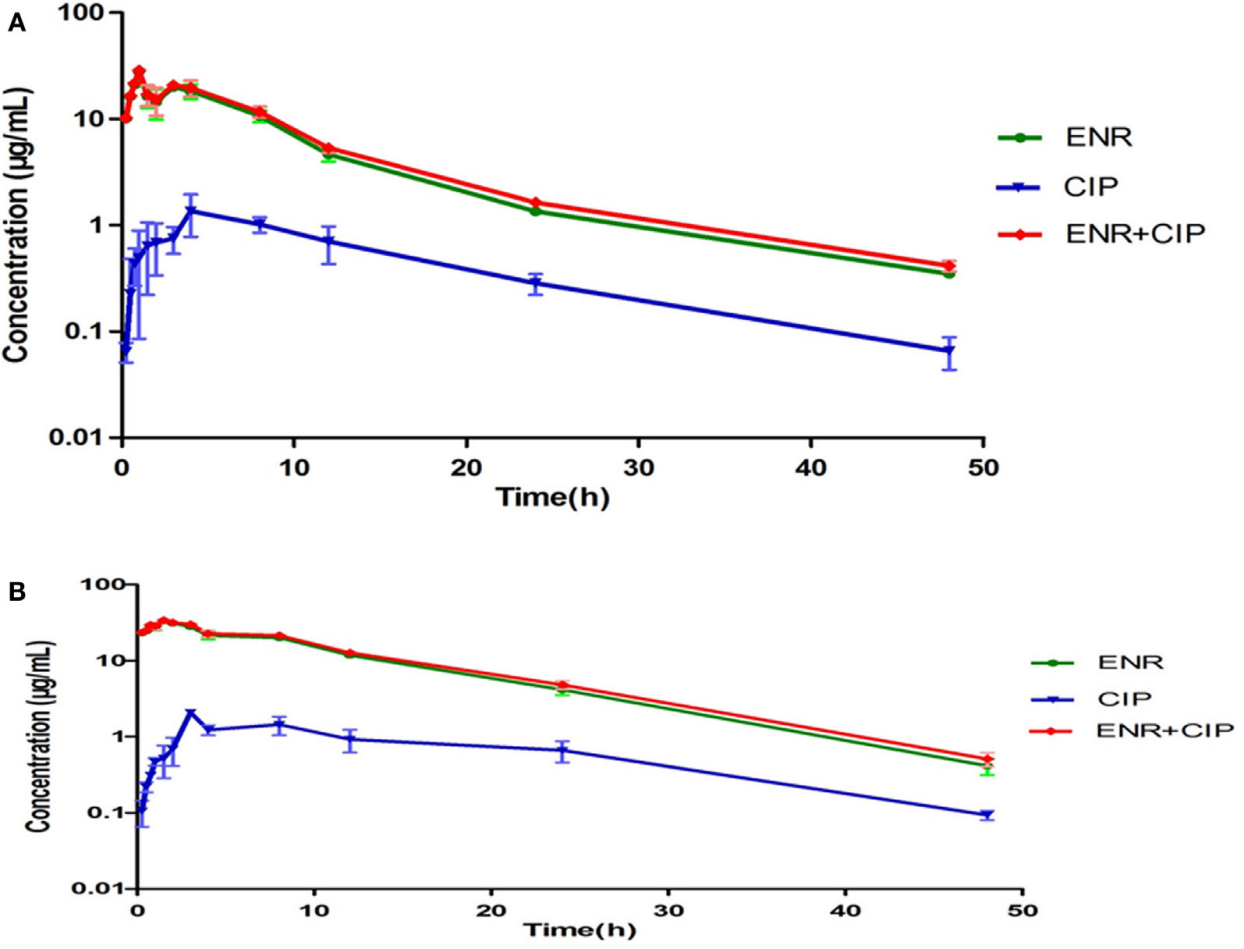
**Concentration-time curves of enrofloxacin and ciprofloxacin in the intestinal contents from healthy chicken (A) and bacterial infected chicken (B)**. The *X*-axis was the time point and *Y*-axis was the drug concentrations. The ciprofloxacin was the metabolite of enrofloxacin in chicken intestinal contents.

The intestinal content concentrations of enrofloxacin and ciprofloxacin were determined after oral administration of the antibiotics to the healthy and infected broilers (Figure 3). After oral administration, plasma and intestinal content concentration of enrofloxacin was best fitted with a one-compartment model, all PK parameters were calculated by WinNonlin 5.2. The PK parameters of enrofloxacin in intestinal content are illustrated in Table 2. In the healthy model, the AUC, *C*_max_, and *T*_max_ were 228.97 μg mL^−1^ h, 21.69 μg mL^−1^, and 1.23 h, respectively. In the infected model the AUC, *C*_max_, and *T*_max_ were 444.86 μg mL^−1^ h, 31.69 μg mL^−1^, and 1.13 h, respectively.

**Table 2:** **Pharmacokinetic parameters of enrofloxacin and ciprofloxacin in intestinal contents after oral administration of enrofloxacin at 10 mg/kg BW to broilers**.

PK parameters (units)	Healthy model			Infected model		
	ENR	CIP	ENR **+** CIP	ENR	CIP	ENR **+** CIP
AUC (μg h/mL)	206.30	18.63	228.97	406.74	31.39	444.86
*T*_max_ (h)	1.18	5.50	1.23	1.06	6.09	1.13
*C*_max_ (μg mL^−1^)	21.07	1.11	21.69	30.88	1.46	31.69
V/F (mL/kg)	413.02	4967.17	403.45	296.59	4392.84	289.02
Cl/F (mL/h/kg)	48.47	536.84	43.67	24.58	318.52	22.48
Ka (h^−1^)	2.79	0.28	2.72	3.66	0.31	3.43
Ke (h^−1^)	0.12	0.11	0.11	0.08	0.07	0.08
T_1/2e_ (h)	5.91	6.41	6.40	8.36	9.56	8.91
T > MIC	39.29	30.08	42.79	49.89	43.59	49.95

### MIC, MBC, MPC, and PAE of Enr and Cip Against Anhui 112

The MIC and MBC of enrofloxacin in MH broth and intestinal contents were similar 0.25 and 0.5 μg/mL, respectively. The MPC value of enrofloxacin and ciprofloxacin against Anhui 112 were 7 and 0.625 μg/mL, respectively. The PAE showed strong concentration-dependent tendencies. These results are presented in Table 3.

**Table 3:** **Antibacterial activity of enrofloxacin and ciprofloxacin against *E. coli in vitro***.

Drug	MIC (**μ**g/mL) Broths and intestinal	MBC (**μ**g/mL) Broths and intestinal	MPC (**μ**g/mL) Broths	PAE		
				Concentration (μg/mL)	Expose (1 h)	Expose (2 h)
ENR	0.25	0.5	7	MIC (0.25)	0.2 h	0.4 h
				2 MIC (0.5)	0.8 h	1.2 h
				4 MIC (1)	1.4 h	1.6 h
CIP	0.125	0.25	0.625	MIC (0.125)	0.2 h	0.4 h
				2 MIC (0.25)	0.8 h	1.4 h
				4 MIC (0.5)	1.5 h	1.6 h

### *In vitro* and *Ex Vivo* Antimicrobial Activity

**Figure 4:**
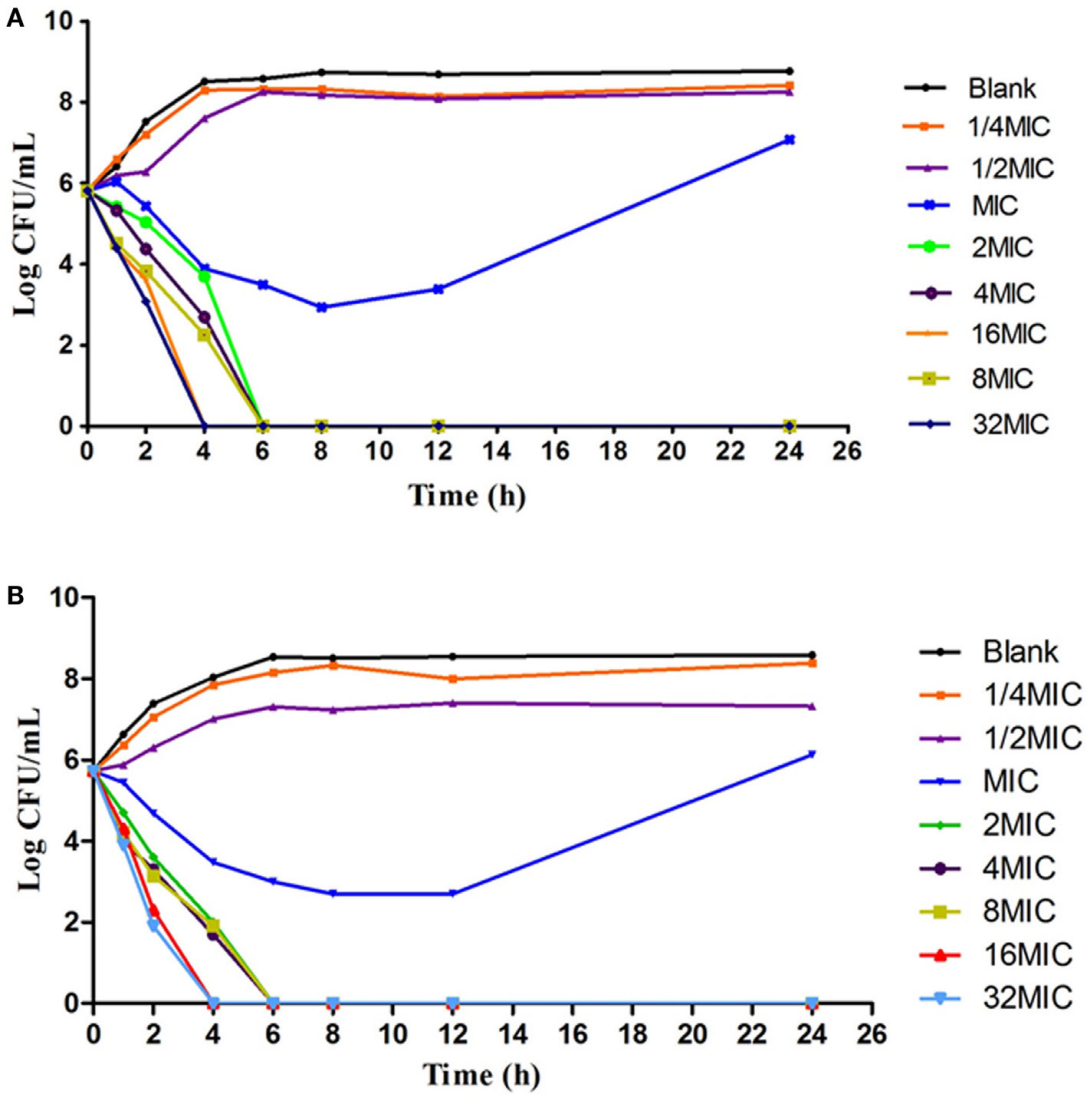
**The *in vitro* kill bacteria curve of enrofloxacin (A) and ciprofloxacin (B) against *E.coli* Anhui112**. The *X*-axis was the time point during the 24-hours incubation and *Y*-axis was the bacterial number under exposure to deferent concentration (1/4 × MIC, 1/2 × MIC….0.32 × MIC) of drug.

According to the MIC values, a series of varying concentrations of enrofloxacin and ciprofloxacin were prepared in MH broth (1/2–32MIC) to ascertain the *in vitro* bactericidal curve against Anhui 112 (Figure 4). The curves are characteristically typical for concentration-dependent antibiotic activity. When exposed to the higher concentration (≥1 × MIC) of enrofloxacin or ciprofloxacin for 6 h, the bacterial CFUs were significantly decreased to undetectable level (<30 CFU). When the bacterial was exposed to 1 × MIC of the two drugs, the growth of bacterial was inhibited at the first 8 h but was rebounded during 8–24 h.

**Figure 5:**
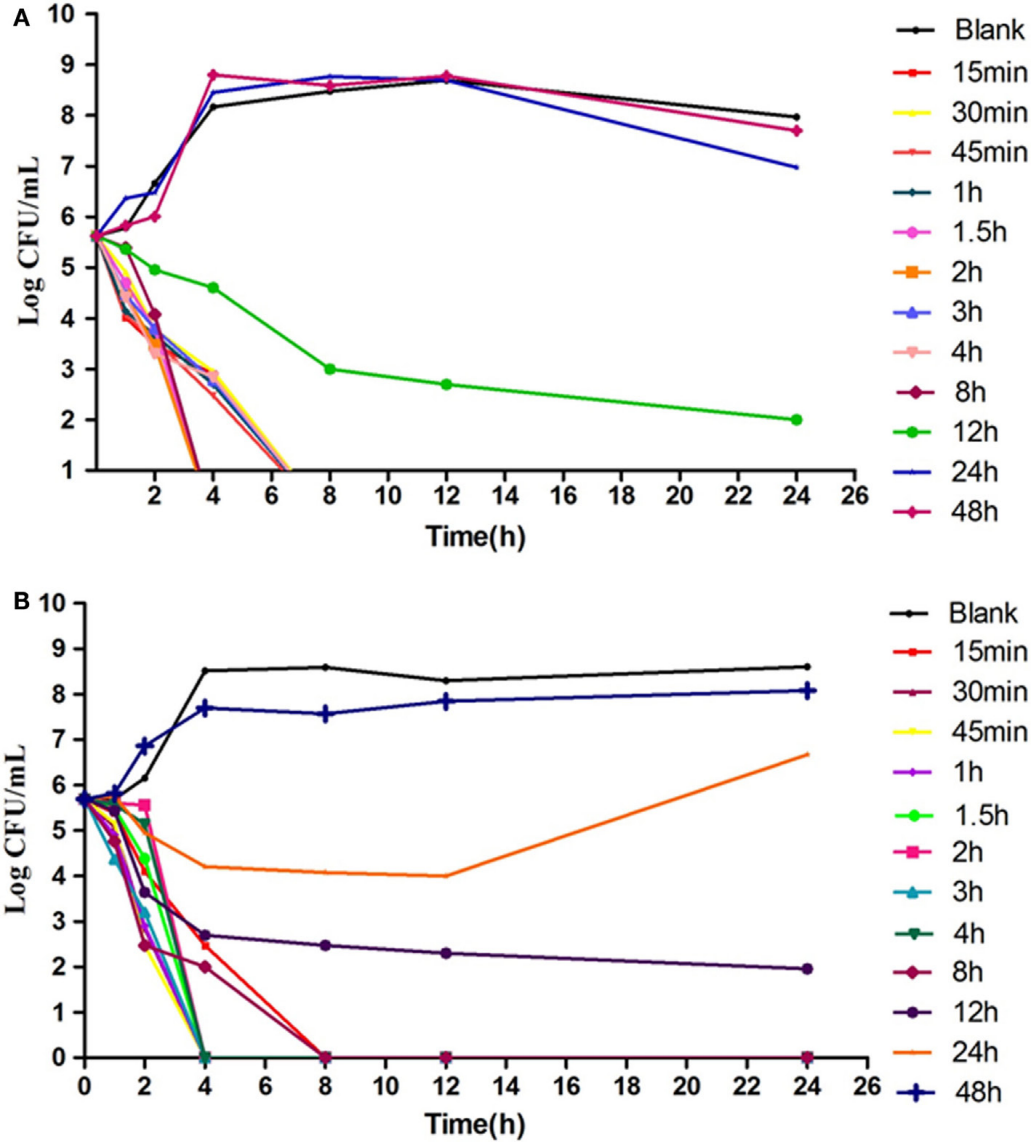
**The*Ex vivo* inhibition curve in the intestinal content from healthy chicken (A) and infected chicken (B)**. The *X*-axis was the time point during the 24-hours incubation and *Y*-axis was the bacterial number under exposure to intestinal samples collected from different time point in the PK study.

The results showed that in an *ex vivo* environment enrofloxacin is typically concentration dependent (Figure 5), consistent with the *in vitro* antibacterial curve. In the high concentration group, the enumerated CFUs changed significantly. After 4-h and 8-h cultures, there were no detectable bacteria in infected broilers.

### PK/PD Integration and Analysis

The integration of *in vivo* PK and *in vitro* PD data of enrofloxacin in intestinal contents showed AUC/MIC_50_ ratios for healthy and infected broilers to be 455.20 and 884.41 h, respectively. The *C*_max_/MIC ratios were 43.38 and 63.38, respectively. Sigmoid *E*_max_ model was utilized to simulate the relationships between *C*_max_/MIC, AUC/MIC, and *ex vivo* antimicrobial efficacy.

### Dosage Determination

According to the dosage equation $X=d\times \frac{AUC_{\text{ex vivo}}/MIC_{\text{tested}}}{AUC_{\text{in vivo}}/MIC_{\text{target}}}$, the optimal dose *X* (mg/kg/d) was calculated. The *d* was the projected dosage (10 mg/kg) in this study. The AUC*_*in vivo*_*/MIC_target_(0.5 μg/mL, the MIC_50_ of enrofloxacin against clinical strains) for infected broilers were 455.20 and 884.41 h, respectively. See Table 4 of *ex vivo* parameters, when *E* = 0, the AUC*_*ex vivo*_*/MIC_test_ (0.25 μg/mL, MIC of enrofloxacin to *E. coli* Anhui112) for healthy and infected broiler were 258.73 and 624.94 h, respectively. When *E* = −3, the AUC*_*ex vivo*_*/MIC_test_ for healthy and infected broiler were 451.35 and 1065.93 h, respectively. When *E* = −4, the AUC*_*ex vivo*_*/MIC_test_ for healthy and infected broiler were 567.39 and 1343.81 h, respectively. Therefore, see Table 5, the doses calculated for bacteriostatic action (*E* = 0) in healthy and infected broilers were 5.6 and 7 mg/kg BW. The doses calculated for bactericidal action (*E* = −3) in healthy and infected broilers were 9.8 and 11.9 mg/kg BW. The calculated for bacterial eradication (*E* = −4) in healthy and infected broilers were 12.4 and 15.1 mg/kg BW. Ultimately, for treatment *E.coli* infection in broiler, the preventive, therapeutic, and bacterial eradication dose of enrofloxacin were chosen as 7.0, 11.9, and 15.1 mg/kg BW.

**Table 4:** **PK/PD parameter of *ex vivo* data after oral administration enrofloxacin in chicken**.

Parameter	Unit	Healthy	Infected
*E*_max_	Log10CFU/mL	2.35	2.91
*E*_0_	Log10CFU/mL	−5.62	−5.69
*E*_max_−*E*_0_	Log10CFU/mL	7.97	8.60
EC_50_	h	348.51	802.26
Slope (N)	–	2.85	2.72
AUC_24_/MIC for bacteriostatic action (*E* = 0)	h	258.73	624.94
AUC_24_/MIC for bactericidal action (*E* = −3)	h	451.35	1065.93
AUC_24_/MIC for bacterial eradication (*E* = −4)	h	567.39	1343.81

**Table 5:** **The different dosages (milligram per kilogram) when MIC of enrofloxacin is 0.5 μg/mL**.

Effect (*E*)	Healthy dosage	Infected dosage
Bacteriostatic action (*E* = 0)	5.6	7.0
Bactericidal action (*E* = −3)	9.8	11.9
Bacterial eradication (*E* = −4)	12.4	15.1

Due to intensive broiler rearing practices, a mixed feeding dosage regimen was also calculated in our study. According to the groups feeding dosing formula D = *d* × *n*/*W*, for 21-day-old broilers, the value of *W* is approximately 120 g/kg/d, *n* is the daily dosing frequency and *D* is group mixed feeding dosage. When the MIC_50_ against clinical strains is 0.5 μg/mL, the optimum dose is 99 mg/kg for mixed feeding.

**Figure 6:**
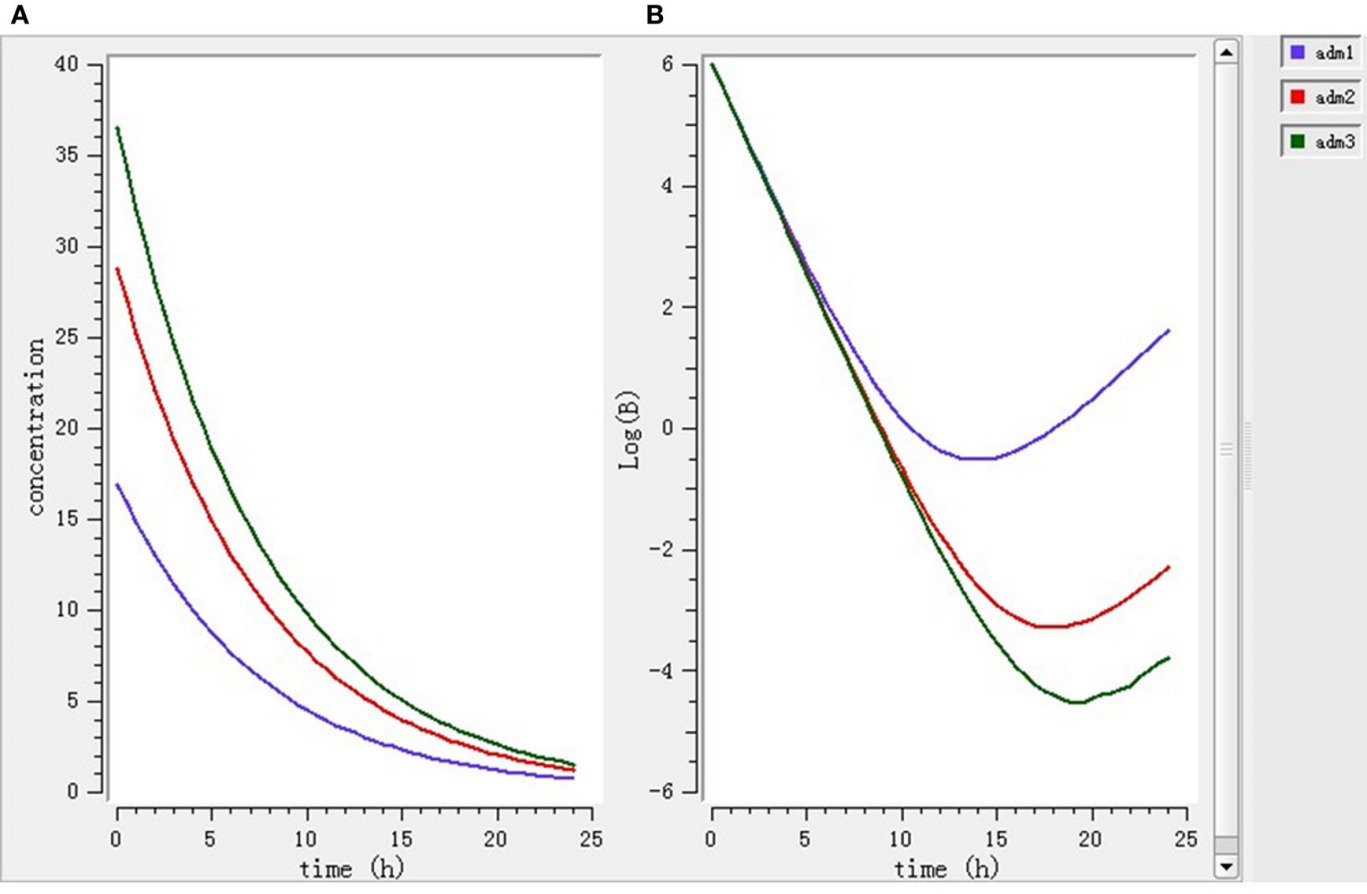
**The pharmacokinetic change (A) and pharmacodynamic change (B) under 1-day single treatment simulated by mlxplore software**. Adm1, 2, and 3 represented 1-day single treatment with 7.0, 11.9, and 15.1 mg/kg enrofloxacin, respectively.

**Figure 7:**
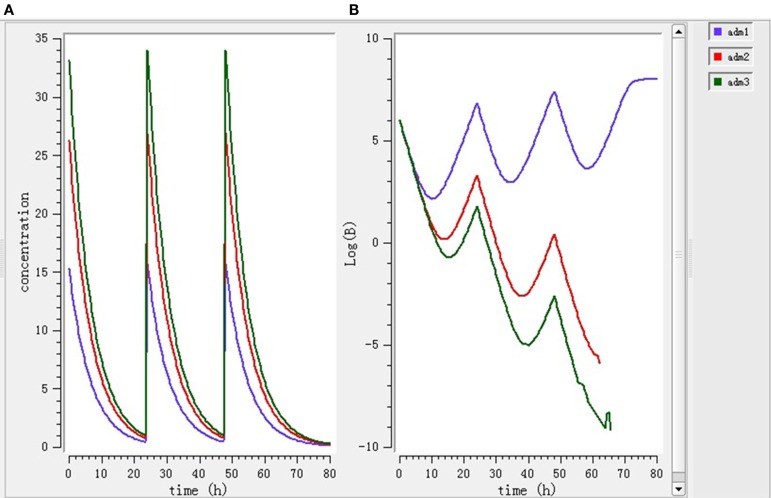
**The pharmacokinetic change (A) and pharmacodynamic change (B) under 3-day treatment simulated by mlxplore software**. Adm1, 2, and 3 represented 3-day daily-treatment with 7,11.9, and 15.1 mg/kg enrofloxacin, respectively.

Using the PK/PD model with the PD parameters derived from *ex vivo* antimicrobial activity, three selected doses (7.0, 11.9, 15.1 mg/kg BW) were separately simulated. As shown in Figure 6, after single dose treatment for 24 h, higher dose (11.9 and 15.1 mg/kg BW) played bactericidal or eradication action during 0–18 h, but the bacteria rebound phenomenon was occurred under the lower dose (7 mg/kg BW) treatment. As shown in Figure 7, after daily-treatment of the three selected doses (7.0, 11.9, 15.1 mg/kg BW) for 3 days, the higher doses (11.9, 15.1 mg/kg BW) exhibited great bactericidal and eradication action; however, the bacterial was not inhibited under lower 3-day treatment of lower dose (7 mg/kg BW).

### Assessment of Bacterial Resistance

Results in Table 6 show that when the drug concentration is higher than 8 μg/mL, drug-resistant strains do not appear; however, when the drug concentration is 2–4 μg/mL, drug resistance strains appear after 36 h. Resistance strains are most easily achieved when the drug concentrations are between 0.25 and 1 μg/mL after 24 h. Similar results were observed between the blank samples (without drug) and the samples whose concentration is 0.125 μg/mL or less. The data indicate resistant strains generally appear after 24 h; therefore, the dosing interval was chosen to be 24 h.

**Table 6:** **The growth of resistant strains after exposed to different drug concentrations**.

Time (h)	B	0.125	0.25	0.5	1	2	4	8
2	N	N	N	N	N	N	N	N
4	N	N	N	N	N	N	N	N
6	N	N	N	N	N	N	N	N
8	+	N	+	N	N	N	N	N
12	+	+	N	N	+	N	N	N
18	+	N	+	N	+	+	N	N
24	++	++	+	++	N	+	N	N
36	++	++	++	+	N	++	++	N
48	++	++	++	++	+	++	++	N

*B, the blank group*.

*N, colony number below 10 CFU/mL*.

*+, colony number between 10 and 100 CFU/mL*.

*++, colony number beyond 100 CFU/mL*.

### Model Validation

Results show that the protection rate of control group was 8.3%, while the protection rate of the experiment group was 83.3%. These data indicate that the dosage regimen chosen has a significant therapeutic effect, suitable for clinical treatment (Table 7).

**Table 7:** **The therapeutic effect of enrofloxacin against colibacillosis**.

Group	Bacterial	Concentration	Sum chickens	Dead chickens	Protection rate
Test	8 mL,10^9^ CFU/mL	11.9 mg/kg BW	12	2	83.3%
Control	8 mL,10^9^ CFU/mL	No treatment	12	11	8.3%

## Discussion

In this investigation, enrofloxacin was orally administered at a clinical recommended effective dose (10 mg/kg BW) in healthy and infected broilers. Although the PK of enrofloxacin in the serum of goats, pigs, calves, horses, and sheep after administration has already been investigated many times (16–19, 30), reports concerning the PK in the intestinal contents are quite few. In this study, enrofloxacin concentrations in intestinal contents were calculated to be 0.35~28.05 μg/mL. These concentrations were much higher than the concentration of enrofloxacin in plasma of healthy broilers, which were between 0.04 and 2.01 μg/mL. According to Slana et al. (24) enrofloxacin concentrations in manure samples, after 15 mg/kg BW orally administered, ranged between 39.2 and 55.0 μg/g. Moraru et al. (31) detected enrofloxacin concentrations in excreta samples ranged between 40.5 and 50.7 μg/g after 3 days orally treatment with 10 mg enrofloxacin/kg BW. Devreese et al. (26) found enrofloxacin concentrations in cloacal samples ranged between 20.1 and 55.7 μg/g after orally treatment of 10 mg enrofloxacin/kg BW. In this study, we found the enrofloxacin concentrations in the intestinal contents were significantly less than previously reported concentrations that maybe due to sample and dosage differences.

The PKs of enrofloxacin in the infected and healthy models were quite different. Higher AUC was obtained in infected broilers (406.7 μg h/mL) when compared to healthy (206.3 μg h/mL) broilers. The differences of the PKs in healthy and infected broilers maybe due to the changes of the physiological and biochemical indexes, such as the change in their body temperature, the decrease of the protein in plasma, decline of blood pressure, anemia, liver dysfunction by acetylation, and so on. The PK parameter in diseased animals is much similar to clinical conditions. As previous research has shown, the metabolic conversion of enrofloxacin to ciprofloxacin is quite different in different animal species for example: 59% in dairy cows, 36% in sheep, 47% in buffalo calves, and 64% in beef steers (32–34).

In this study, the ratio of ciprofloxacin AUC (31.39) and enrofloxacin AUC (406.74) is 7.71% after oral administration in infected broilers. Differing from other animal species, the biotransformation of enrofloxacin to ciprofloxacin in poultry is limited to only 5–10%, and the ciprofloxacin concentrations are always below the LOQ. (23, 35, 36). The metabolite ciprofloxacin usually has antimicrobial activity, so it is quite important to evaluate the combined action of the enrofloxacin and ciprofloxacin (37). As such, the sum of the concentrations of two drugs should be evaluated by the PK/PD integration and modeling.

The MIC results suggest a bimodal distribution and the 349 strains with MIC ranging between 1 and 64 μg/mL indicates the high increase of antibiotic resistance. Compared to other studies on APEC and AFEC strains (38), 28.5% strains with MIC between 0.5 and 1 μg/mL were considered intermediately resistant, confirming the trend of increasing resistance. In recent years, it has been suggested that the optimization of dosing regimen not only for the treatment but also for reducing antimicrobial resistance development. MIC is used as an *in vitro* reference value to predict the antimicrobial efficacy and potency of a drug. The MIC values in broth and biological fluids reported in previous research (39) shows that the serum inhibitory activity is reduced for most fluoroquinolones. In this study, the MIC values of the pathogen *E. coli* (Auihui 112) for enrofloxacin in intestinal contents and broth both are 0.25 μg/mL, which indicates that enrofloxacin has the same antimicrobial activity in broth and intestinal contents. In our study, the *in vitro* and *ex vivo* time-kill curves indicate that enrofloxacin rapidly eliminates *E. coli*. Rapid inhibition and killing of bacteria exhibited by *ex vivo* inhibition curves is consistent with *in vitro* time-kill data and the previous studies published for danofloxacin and marbofloxacin (39, 40). The time-kill curves seem to indicate that enrofloxacin is concentration dependent. The data from the current study show that bacterial counts will increase after 24-h incubation a time when the antibiotic concentration is lower. These data confirms findings in previous publications stating that MIC alone does not offer enough information about the efficacy of therapy (21).

Enrofloxacin shows a concentration-dependent killing activity like other fluoroquinolone drugs (41). The main PK/PD parameters for concentration-dependent antimicrobials are *C*_max_/MIC and AUC/MIC, the AUC/MIC ratio is a more important surrogate for the PK/PD analysis (42).

The breakpoint of *C*_max_/MIC ≥ 10 and AUC_24_/MIC ≥ 125 is an indicator for the success of therapy and preventing the emergence of resistance of fluoroquinolones (43). *In vivo* AUC_24_/MIC_50_ are 455.20 and 887.41, respectively, for healthy and infected broilers at the dosage of 10 mg/kg. The values of *ex vivo* AUC/MIC obtained for bactericidal action in intestinal contents from healthy and infected broilers is 451.35 and 1065.93, respectively. The enrofloxacin concentrations in intestine are detected in much higher concentrations than in plasma; which would potentially lead to an eradication of the *E. coli* in intestine. In this study, based on the MIC_50_ = 0.5 μg/mL, values of *C*_max_/MIC (43.12) and AUC_24_/MIC (455.20) both values exceed the predictive effective values. These values are comparable with the values of *C*_max_/MIC (38) and AUC_24_/MIC (293) obtained from buffalo calves (44).

Pharmacokinetic/pharmacodynamic modeling serves as a bridge between PK and PD and has been considered as a promising approach (4, 8, 20). Results obtained from this study based on the PK/PD integration suggest that when the MIC_50_ of enrofloxacin is 0.5 μg/mL, for bacteriostasis, bactericidal, and bacterial eradication in infected broilers, the dosage should be 7.0, 11.9, and 15.1 mg/kg BW., respectively. Results from professional software used to simulate different dosage regimens for treatment show that a 3-day treatment of 11.9 mg/kg every 24 h should be efficient against *E. coli* (Anhui 112) in broilers. Therefore, the dose of 11.9 mg/kg BW. should be effective for treating colibacillosis when the MIC ≤ 0.5 μg/mL.

Antimicrobial resistance is a principal threat for both animals and humans. Using PK/PD modeling choosing an appropriate therapeutic schedule is a suitable strategy to reduce antimicrobial resistance without influencing the treatment effect. Previous studies show that both AUC/MIC and *C*_max_/MIC were related to emergence of resistance in *Pseudomonas aeruginosa* (45). In recent years, the drug resistance risk steadily increases rapidly. However, when the MIC is above the MPC in a wild population, the probability that it will undergo two resistant mutations is very low. It has been proposed that the AUC_24_/MPC ratio could help as an indicator of drug exposure that stops the selection of drug-resistant mutants (46, 47). It has been proven that an AUC/MPC ratio that in the range of 9 and 12 could prevent the selection of resistant mutants for marbofloxacin in *E. coli* when the inoculum sizes was 10^5^ and 10^7^ CFU/mL (48). In this study, the AUC/MPC of enrofloxacin in healthy and infected broilers was 29.4 and 58.1, respectively. Indicating that the dosage used in this study potentially can prevent the selection of enrofloxacin resistant mutants.

## Conflict of Interest Statement

The authors declare that the research was conducted in the absence of any commercial or financial relationships that could be construed as a potential conflict of interest.
